# Interactions of human prostatic epithelial cells with bone marrow endothelium: binding and invasion

**DOI:** 10.1054/bjoc.2001.1804

**Published:** 2001-05

**Authors:** L J Scott, N W Clarke, N J R George, J H Shanks, N G Testa, S H Lang

**Affiliations:** CRC Experimental Haematology Group, Paterson Institute for Cancer Research, Christie Hospital NHS Trust, Wilmslow Road, Manchester, M20 4BX, UK; Department of Surgery andDepartment of Histopathology, Christie Hospital NHS Trust, Wilmslow Road, Manchester, UK; Department of Urology, Withington Hospital, Manchester, UK, andYCR Cancer Research Unit, Biology Department, The University of York, York, YO10 5YW

**Keywords:** prostate cancer, prostate epithelia, bone marrow endothelium, metastasis

## Abstract

Prostate cancer shows a propensity to form secondary tumours within the bone marrow. Such tumours are the major cause of mortality in this disease. We have developed an in vitro system to study the binding of prostate epithelial cells to bone marrow endothelium (BME) and stroma (BMS). The metastatic prostate cancer cell line, PC3 (derived from a bone metastasis), was seeded onto confluent layers of BME and its binding characteristics compared to human umbilical vein endothelial cells (HUVEC), lung endothelium (Hs888Lu) and BMS. The PC3 cell line showed significantly increased binding to BME (*P*< 0.05) compared to endothelium derived from HUVEC and lung or BMS with maximal binding occurring at 1 h. Following pre-incubation with a β1 integrin antibody PC3 binding to BME was inhibited by 64% (*P*< 0.001). Antibodies directed against the integrins β4, α2, α4, α5 and the cellular adhesion molecules P-selectin, CD31, VCAM-1 and sialy Lewis X showed no effect on blocking PC3 binding. Primary prostatic epithelial cells from both malignant (*n* = 11) and non-malignant tissue (*n* = 11) also demonstrated equivalent levels of increased adhesion to BME and BMS compared to HUVEC, peaking at 24 h. Further studies examined the invasive ability of prostate epithelial cells in response to bone marrow endothelium using Matrigel invasion chamber assays. In contrast to the previous results, malignant cells showed an increase (1000 fold) in invasive ability, whilst non-malignant prostate epithelia did not respond. We have shown that both malignant and non-malignant prostate epithelial cells can bind at equivalent levels and preferentially to primary human bone marrow endothelium in comparison to controls. However, only malignant prostate epithelia show increased invasive ability in response to BME. © 2001 Cancer Research Campaign www.bjcancer.com


British Journal of Cancer (2001) **84**(10), 1417–1423
© 2001 Cancer Research Campaign
doi: 10.1054/ bjoc.2001.1804, available online at http://www.idealibrary.com on IDEAL®

http://www.bjcancer.com

### Interactions of human prostatic epithelial cells with bone marrow endothelium: binding and invasion

**LJ Scott[1], NW Clarke[2], NJR George[3], JH Shanks[4], NG Testa[1] and SH Lang[5]**

[1]CRC Experimental Haematology Group, Paterson Institute for Cancer Research, Christie Hospital NHS Trust, Wilmslow Road, Manchester, M20 4BX, UK; [2]Department of Surgery and [4]Department of Histopathology, Christie Hospital NHS Trust, Wilmslow Road, Manchester, UK; [3]Department of Urology, Withington Hospital, Manchester, UK, and [5]YCR Cancer Research Unit, Biology Department, The University of York, York, YO10 5YW

**Summary** Prostate cancer shows a propensity to form secondary tumours within the bone marrow. Such tumours are the major cause of mortality in this disease. We have developed an in vitro system to study the binding of prostate epithelial cells to bone marrow endothelium (BME) and stroma (BMS). The metastatic prostate cancer cell line, PC3 (derived from a bone metastasis), was seeded onto confluent layers of BME and its binding characteristics compared to human umbilical vein endothelial cells (HUVEC), lung endothelium (Hs888Lu) and BMS. The PC3 cell line showed significantly increased binding to BME ($P < 0.05$) compared to endothelium derived from HUVEC and lung or BMS with maximal binding occurring at 1 h. Following pre-incubation with a β1 integrin antibody PC3 binding to BME was inhibited by 64% ($P < 0.001$). Antibodies directed against the integrins β4, α2, α4, α5 and the cellular adhesion molecules P-selectin, CD31, VCAM-1 and sialy Lewis X showed no effect on blocking PC3 binding. Primary prostatic epithelial cells from both malignant ($n = 11$) and non-malignant tissue ($n = 11$) also demonstrated equivalent levels of increased adhesion to BME and BMS compared to HUVEC, peaking at 24 h. Further studies examined the invasive ability of prostate epithelial cells in response to bone marrow endothelium using Matrigel invasion chamber assays. In contrast to the previous results, malignant cells showed an increase (1000 fold) in invasive ability, whilst non-malignant prostate epithelia did not respond. We have shown that both malignant and non-malignant prostate epithelial cells can bind at equivalent levels and preferentially to primary human bone marrow endothelium in comparison to controls. However, only malignant prostate epithelia show increased invasive ability in response to BME. © 2001 Cancer Research Campaign http://www.bjcancer.com

**Keywords**: prostate cancer; prostate epithelia; bone marrow endothelium; metastasis

The second commonest cause of cancer death in the Western world is attributed to prostate cancer (Jensen et al, 1990). It is well documented that prostatic carcinoma shows a predilection to metastasize to the bone marrow (Jacobs, 1983). Metastatic prostate cancer remains an incurable disease and as such, is a massive clinical problem. There is clearly a need to elucidate the factors underlying the spread of prostate cancer, particularly to the skeleton.

It has been suggested that the bone marrow microenvironment is conducive to the growth of prostate cancer cells, which non-selectively enter the bone marrow from the circulation (Galasko, 1981; Jacobs, 1983; Paget, 1989; Body, 1992). However, the strikingly consistent pattern of prostate metastasis within the red marrow suggests that this process may in fact be regulated (Fidler et al, 1978). The mechanism of metastasis is a complex multi-step process that is not fully understood. One critical step in this mechanism may be the attachment to and extravasation through endothelial barriers by malignant cells possibly leading to selective metastatic sites. Tumour cell binding to endothelium involves two distinct steps, an initial docking step mediated via lectin–carbohydrate interactions followed by an integrin-mediated locking step (Honn and Tang, 1992). Several endothelial and tumour adhesion molecules have been associated with metastasis. In particular the integrins β1, α2 and α5 have been shown to be expressed by prostate epithelial cells and bone marrow cells (Soligo et al, 1990; Nagle et al, 1994; Rokhlin and Cohen, 1995). The carbohydrate sialyl Lewis X has also been associated with breast and lung cancer metastasis and its ligand P selectin is found on endothelial cells (Soligo et al, 1990). Some lung, brain, liver and ovary metastatic tumour cells have been demonstrated to bind selectively to endothelial cells isolated from lung, brain, liver and ovary respectively (Nicolson and Winkelhake, 1975; Auerbach et al, 1987). These studies suggest an active regulatory role for the endothelium in metastasis (Zetter, 1990).

We have shown previously that primary prostatic epithelia from both benign and malignant tissue show an accelerated growth rate within bone marrow stroma compared to control stroma (Lang et al, 1998) and also that integrin α2β1 is a major contributor to the binding of primary prostatic epithelial cells to bone marrow stroma (Lang et al, 1997). This pattern of primary prostatic epithelial cell adhesion (α2β1) is mimicked by the prostate cell line, PC3 (Kostenuik et al, 1996) and our experiments were therefore conducted with this cell line. These studies have now been extended to develop a model to investigate the interactions of prostatic epithelial cells (primary and cell lines) with the bone marrow endothelium.

*Received 16 August 2000*
*Revised 13 February 2001*
*Accepted 15 February 2001*

*Correspondence to:* LJ Scott

### MATERIALS AND METHODS

### Materials

General chemicals were purchased from Sigma (Poole, UK). Tissue culture media and supplements were obtained from Gibco

Ltd (Paisley, UK) with the exception of endothelial growth media (EGM-2) from Bio Whittaker (Wokingham, UK) and HAMS-F12 from PAA Laboratories (Linz, Austria). Fetal calf serum (FCS) was purchased from Advanced Protein Products (Briely Hill West, UK), horse serum from Autogen Bioclear (Wiltshire, UK) and Worthington Collagenase from Lorne Laboratories (Twyford, UK).

### Antibodies

Mouse monoclonal anti-vimentin, anti-pan-cytokeratin (clone C11), anti P selectin and anti-von-Willebrand factor antibodies were all obtained from Sigma. Mouse monoclonal anti β1, α2, α4, α5 integrins and sialy Lewis X (CD15s) antibodies were from Becton Dickinson (Oxford, UK). Anti-CD31, mouse IgG, rabbit anti-mouse horse-radish peroxidase and swine anti-rabbit horse-radish peroxidase were all from Dako (High Wycombe, UK). Monoclonal anti β4 integrin was from Serotec (Oxford, UK) and rabbit anti-human cytokeratin from Biogenesis (Poole, UK).

### Tissue collection

Bone marrow aspirates were collected from patients undergoing surgery for benign diseases after informed consent had been obtained. Prostate tissue was collected from patients with benign prostatic hyperplasia or adenocarcinoma of the prostate undergoing transurethral resection for bladder outflow obstruction. Each individual prostatic chip was halved, half was sent for histological analysis for the presence/absence of tumour cells; the remaining was used for tissue culture.

### Bone marrow stromal culture

Long-term bone marrow stroma (BMS) cultures were established according to the protocol of Coutinho et al (1993). In brief, bone marrow samples were depleted of red blood cells by using a 0.1% (w/v) methylcellulose solution. The remaining cells were then seeded at $2 \times 10^6$ cell ml$^{-1}$ in bone marrow growth medium (Iscove's modified Dulbecco's medium (350 mOsm), 10% FCS, 10% horse serum, $5 \times 10^{-7}$ M hydrocortisone and 1% antibiotic/antimycotic solution). The cultures were grown at 33˚C in 5% $CO_2$ in air. Growing cultures were fed weekly by removal of half the medium followed by replacement with fresh growth medium. After approximately 4 weeks of growth confluent haemopoietically active cultures were observed (containing a heterogeneous mix of cells including adipocytes, macrophages, endothelial and fibroblasts).

### Bone marrow endothelial cell isolation and culture

Bone marrow endothelial cells (BME) were isolated by modifying the technique of Masek and Sweetenham (1994). Briefly, bone marrow aspirates (10–20 ml) were diluted (1:1 v/v) into Dubecco's modification of Eagles medium (DMEM) containing heparin (30–40 U ml$^{-1}$). The marrow was then filtered through a 70 μm mesh strainer, washed 2× with Hanks balanced salt solution (HBSS). Red cells were lysed using ammonium chloride lysis buffer. The remaining cells were then centrifuged at 200 ***g*** for 5 min and re-suspended in PBS (10 mM phosphate buffer (pH 7.3), 137 mM NaCl and 3 mM KCL) containing 1% bovine serum albumin (BSA) and 5 mM EDTA (wash buffer). Magnetic Dyna beads (Dynal, Wirral, UK) were coupled to Ulex europaeus agglutinin-1 (UEA-1) (Sigma, Poole, UK) as described by Jackson et al (1990). The coated dyna beads were mixed with the cell suspension at a bead to cell ratio of 5 beads per endothelial cell (assuming endothelial cells comprise approximately 1% of the total cell count (Masek and Sweetenham, 1994) for 10 min at 4˚C on a rotary mixer. The BME cells bound to the UEA-1 coated beads were washed 5× by resuspending in 5 ml of wash buffer and mixing for 1 min followed by separation using a magnetic particle concentrator (Dynal, Wirral, UK). Finally the isolated endothelial cells were re-suspended in endothelial plating media containing 5% FCS and seeded into a 12.5 ml fibronectin coated (50 μg ml$^{-1}$) tissue culture flask. After 24 h the media was removed and replaced with EGM-2 containing 5% FCS. The cells were grown to confluence at 37˚C in 5% $CO_2$ in air. Cells were passaged up to 4 times using trypsin for endothelial cell cultures (Sigma, Poole, UK). The endothelial nature of the cells was confirmed by immunohistochemical staining for known endothelial markers.

### Prostatic epithelial and fibroblast cell culture

Prostatic epithelia were isolated and characterized as described by Lang et al (1998). Briefly, prostate specimens were minced and incubated over night in collagenase (200 U ml$^{-1}$) in RPMI 1640 supplemented with 5% FCS on a shaking platform at 37˚C. The solution was then broken up by pipetting and washed once in RPMI 1640 medium with 5% FCS at 800 ***g*** for 5 min. The pellet was then resuspended in 0.1% (w/v) trypsin and incubated for a further 30 min at 37˚C on a shaker. The final digest was washed 3 times then centrifuged at 360 ***g*** for 1 min to produce a pellet enriched for epithelia. The epithelial cells were resuspended in serum-free Keratinocyte media (Gibco) and passed through a 40 μm cell sieve to give a single cell suspension. The epithelial cells were frozen in FCS and 10% DMSO until use. Epithelial cells have been shown previously to contribute to between 30–80% of the cell population as assessed by fluorescent-activated cell sorting of cytokeratin positive cells (Lang et al, 1998) (contaminating cell types were mainly blood cells with a few remaining fibroblasts). Subsequent growth of epithelia was favoured by using serum-free keratinocyte media.

### Culture of cell lines

Human umbilical vein endothelial cells (HUVEC) and lung microvascular cells (Hs888Lu) were obtained from the European Collection of Animal Cell Cultures (Porton Down, UK). HUVEC and Hs888Lu cells were cultured in EGM-2. Cells were passaged using trypsin for endothelial cells, HUVEC cells were used up to passage 8 and Hs888Lu cells up to passage 4. The prostatic carcinoma cell line, PC3, was routinely cultured in HAMS-F12 supplemented with 2 mM glutamine with 7% FCS and the normal prostate cell line, PNT2-C2 (Berthon et al, 1995), in RPMI-1640 containing 2 mM glutamine with 10% FCS.

### Binding assay

Endothelium ($1 \times 10^5$ cells ml$^{-1}$) or stroma (confluent T-12.5 plate$^{-1}$) were seeded into 96-well plates (200 μl well$^{-1}$) and grown to confluence. Once confluent half the media was removed and replaced with 100 μl of Hams-F12 with and without the addition of 5000 PC3 or primary prostatic epithelial cells. The epithelial

 © 2001 Cancer Research Campaign cells were allowed to bind for a given length of time before removal of any unbound cells by washing 3 times with PBS. Subsequently, the plates were fixed with a mixture of methanol:acetone (1:1, v:v) for 20 min at –20˚C. The fixative was then removed and the plates allowed to air dry. Epithelial cell binding was assessed by staining for cytokeratin, as follows: endogenous peroxidases were blocked by the addition of 3% $H_2O_2$ in PBS for 30 min at room temperature. The plate was subsequently washed with PBS/0.05% Tween 20 (PBS-T). Non-specific binding sites were blocked with 20% rabbit serum in 1% BSA/PBS for 1 h at room temperature. After 3 washes with PBS-T, the cells were then incubated with a mouse anti-human pan cytokeratin antibody diluted 1:3200 (v:v) for 1 h at room temperature. Following washing, a secondary horse-radish peroxidase-linked rabbit anti-mouse antibody was added and allowed to bind for a further 1 h. The plate was then washed once with PBS-T, once with $dH_2O$ and once with ABTS buffer (Boehringer Mannheim, Germany) followed by the addition of the ABTS substrate (Boehringer Mannheim, Germany). Finally, the substrate was developed for 45 min at room temperature and the optical density read at 405 nm. The number of bound epithelial cells was expressed as the optical density of cytokeratin fluorescence, which was shown to be linearly related (results not shown).

### Inhibition studies

PC3 cells were pre-incubated with antibodies against β4, β1, α2, α4 and α5 integrins and sialy Lewis X for 30 min at 37˚C. Control cells were incubated with mouse IgG. All antibodies were used at 20 $\mu g\ ml^{-1}$ (Lang et al, 1997). The cells were then added to confluent monolayers of BME at a density of $5 \times 10^4$ $well^{-1}$. Binding was allowed to take place for 1 h at 37˚C. The plates were then washed 3× with PBS and fixed with methanol:acetone (1:1, v:v) for 20 min at –20˚C. BME were also pre-treated with antibodies against P selectin, CD31 and VCAM-1 (20 $\mu g\ ml^{-1}$) for 30 min. $5 \times 10^4$ PC3 $well^{-1}$ were then added and incubated for 1 h. The plates were then washed and fixed as stated above. PC3 binding was measured using the binding assay with the following modifications; non-specific binding sites were blocked with 5% human serum in 1% BSA/PBS, the mouse anti-human cytokeratin antibody was substituted by a rabbit anti-human cytokeratin antibody (1:250) to prevent any cross-reactivity with the inhibitory antibodies and the secondary antibody was replaced with a swine anti-rabbit horse-radish peroxidase antibody (1:1000). The plates were then developed as before.

### Immunocytochemistry

Cells to be stained were grown in 8-well glass chamber slides until confluent. The cells were then fixed with methanol:acetone (1:1, v:v) at –20˚C for 20 min. All staining was performed at room temperature in a humidified chamber. Non-specific binding sites were blocked by the addition of 20% rabbit serum or swine serum (depending on the origin of the secondary antibody) in 1% BSA/PBS for 1 h. Wells were subsequently washed 3 times with PBS. Primary antibodies were then added and allowed to bind for 1 h. Endogenous peroxidases were inhibited with 1% $H_2O_2$ in PBS for 10–15 min followed by 3 washes with PBS. Rabbit anti-mouse or swine anti-rabbit biotinylated secondary antibodies (depending on the origin of the primary antibody) were then added (1:400) for 40 min. Following washing with PBS, Vectorstain ABC-HRP (Vector Laboratories, Peterborough, UK) was added and allowed to bind for 15 min then washed. Positively stained cells were then observed by the addition of DAB (Sigma, Poole, UK) for 5–15 min. The slides were washed in $dH_2O$ and counter stained with 1% Gills haematoxylin.

### Invasion assay

Invasion chambers were prepared by coating cell culture inserts (8 µm pore size, Becton Dickinson) with 100 µl of Matrigel (Becton Dickinson) diluted 1:45 (v:v) with Dulbecco's modified Eagles Medium (DMEM, Life Technologies). The inserts were incubated over night at 37˚C. Growth media was aspirated from confluent endothelial/stromal cultures grown in 24-well plates and replaced with 1 ml DMEM supplemented with 0.1% BSA. Matrigel-coated inserts were placed over the endothelial/stromal cultures. Epithelial cells (PC3, PNT2-C2 or BPH) were prepared in DMEM supplemented with 0.1% (w:v) BSA to a cell concentration of $2 \times 10^5$ cells $ml^{-1}$ and 0.5 ml of this cell suspension was added to each insert. Control wells were prepared which contained no endothelial/stromal monolayer. Each experiment was carried out in triplicate. The invasion assay was left for 18 h after which cells that had not invaded were removed from the top of the insert by scrubbing with a cotton bud. The inserts were then fixed in methanol for 10 min and then stained with 0.1% (w:v) crystal violent (Sigma). Cells that had invaded to the underside of the insert were then counted according to the manufacturer's instructions.

### Statistics

All assays were compared by use of the two-tailed Student's *t*-test. A threshold of significance was set at $P < 0.05$.

### RESULTS

### Characterization bone marrow endothelial cells

The cells showed the characteristic spindle-shaped morphology described previously by Masek and Sweetenham (1994). UEA-1 coated magnetic beads can be seen still attached to many of the endothelial cells (Figure 1A, mouse IgG1 negative control). The endothelial nature of the cultured cells was confirmed by staining for von-Willebrand factor (Figure 1B) the major marker for endothelial cells. Cultures from up to 4 passages were used for assays. Table 1 summarizes the staining characteristics of bone marrow endothelium compared to human umbilical vein endothelial and PC3 cells.

### Binding assay

An assay to measure the binding characteristics of prostatic epithelial cells to endothelium or stroma was developed. The assay was initially established using the prostatic cell line PC3. This particular cell line was chosen as it was derived from a human prostatic bone marrow metastasis and has been shown to metastasize in about 30% of cases when injected subcutaneously into nude mice (Shevrin et al, 1989). It has also been shown to interact with bone marrow stroma in an identical manner to primary prostatic epithelial cells (Kostenuik et al, 1996; Lang et al, 1997).

Binding of PC3 cells to HUVEC, BMS (positive control) or BME was measured over 4 h after which levels began to plateau

© 2001 Cancer Research Campaign 

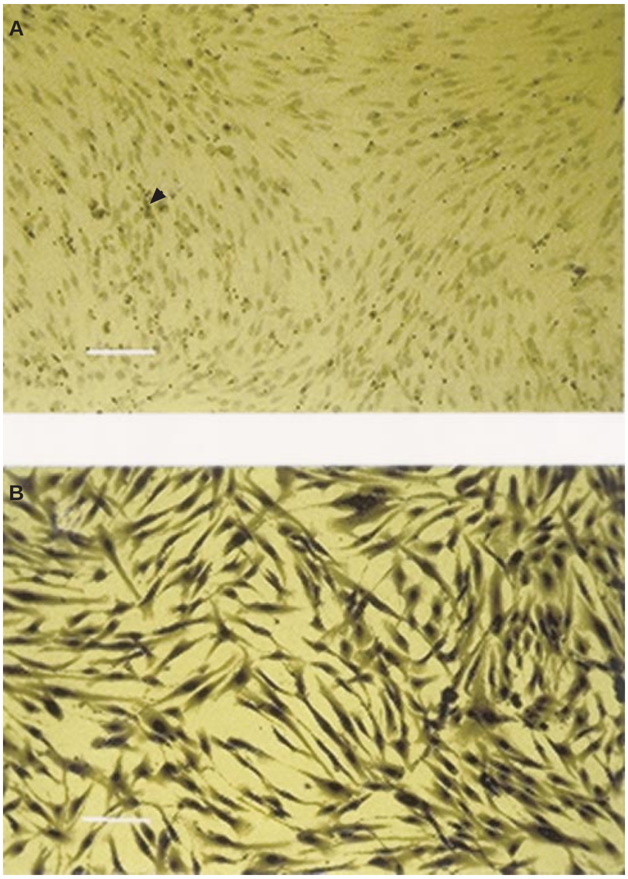

**Figure 1** Immunohistochemical staining of cultured bone marrow endothelial cells. (**A**) Negative control (mouse IgG1). UEA-1 coated magnetic beads can be seen still attached to some of the cells, arrowhead, (**B**) von Willebrand factor. Bar corresponds to 100 μm off (binding was not measured over a longer time course as it was felt that cell division of bound epithelial cells would lead to falsely high levels being measured). The majority of cells bound within the first 60 min. Therefore this time point was chosen to compare binding levels in future experiments (Figure 2). Both the rate and level of PC3 binding was higher to BME compared to HUVEC with a significant difference ($P$ = 0.006) already demonstrated after 15 min incubation. The rate of adhesion to BMS was slower than that observed to BME. A significant increase in the binding of PC3 cells to BME versus BMS was found at 15 min ($P$ = 0.025). After 30 min there was no difference between the levels of adhesion. The binding of PC3 cells to different endothelium or stroma

**Table 1** Immunohistochemical staining of resting cells +++ = very strong staining, ++ = strong staining, + = weak staining, +/ − = positive in some of the cells and − = negative

| Antibody | BME | HUVEC | PC3 |
|---|---|---|---|
| von Willebrand factor | +++ | +++ | – |
| P selectin | +++ | + | – |
| CD31 | +/– | +/– | – |
| Vimentin | +++ | ++ | +++ |
| Pan cytokeratin | – | – | ++ |
| UEA–1 | ++ | ++ | – |

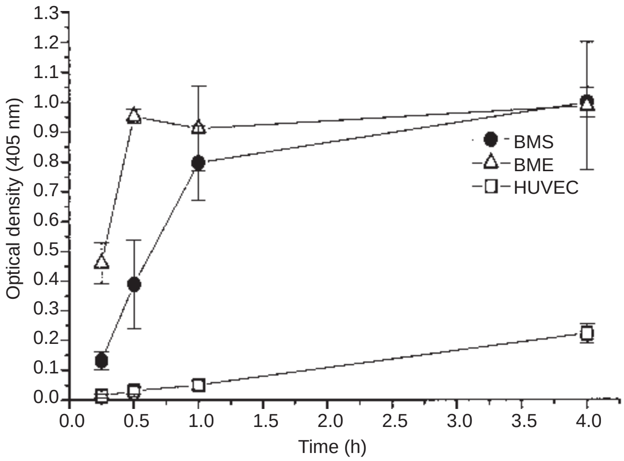

**Figure 2** Time course of PC3 binding to BME, BMS or HUVEC. 5 × 10[4] PC3 cells were added per well. PC3 binding is represented by optical density of cytokeratin fluorescence. Data represent the mean of 3 experiments (6 replicas per assay), bars correspond to standard error of mean following a 1 h incubation is summarized in Figure 3. They showed significantly greater adhesion to bone marrow endothelium and bone marrow stroma compared to endothelium derived from human umbilical vein endothelial cells ($P$ = 0.008 and 0.009, respectively). Binding to the lung endothelial cells (Hs888Lu) was also significantly higher than to HUVEC ($P$ = 0.00008). Endothelial binding of the prostatic cell line, PNT2-C2, derived from normal prostate tissue also showed a similar pattern to that of PC3 cells (data not shown).

To establish which adhesion molecules may be involved in the binding of PC3 cells to BME, inhibitory antibodies against P-selectin, CD 31 and VCAM-1 were pre-incubated with bone marrow endothelial cells prior to performing a binding assay. Alternatively, the PC3 cells were pre-incubated with antibodies directed against β4, β1, α2, α5, α4 integrins and sialy Lewis X before carrying out a binding assay to BME. Figure 4 shows the effect of using antibodies raised against adhesion molecules on

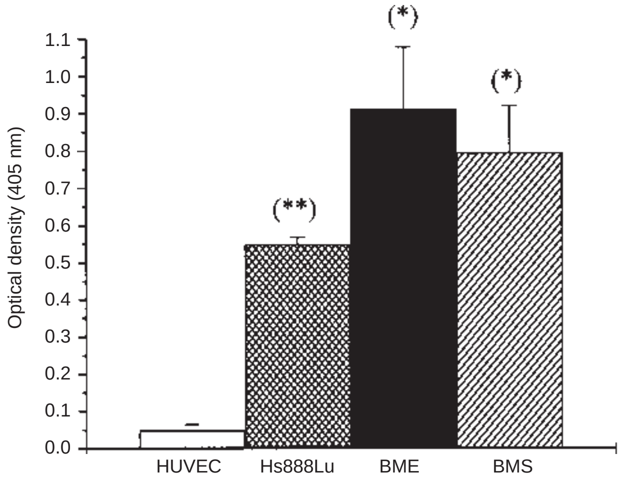

**Figure 3** Comparison of PC3 cells binding (5 × 10[4] per well) to various endothelium or stroma after 1 h incubation. Data corresponds to mean of 3 experiments (6 replicas per assay), bars represent standard error of mean. *= $P$ <0.05 and **= $P$ <0.001 (compared to binding to HUVEC)

 © 2001 Cancer Research Campaign

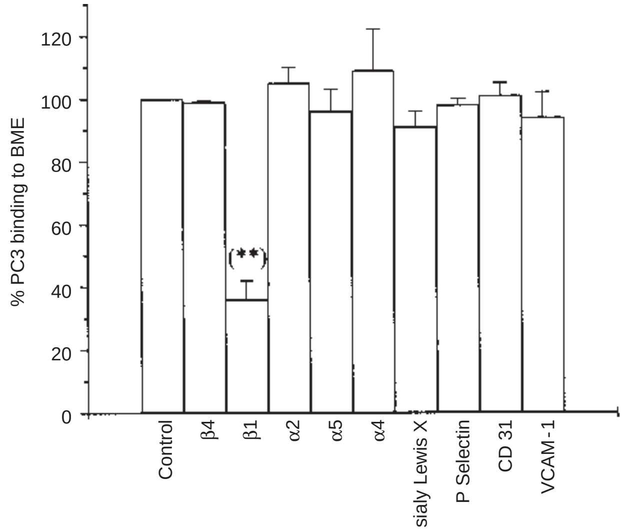

**Figure 4** Inhibition of PC3 binding to BME following pre-incubation (for 30 min) with antibodies against cell adhesion molecules. Data demonstrates mean for 3 experiments (3 replicas per assay), bars correspond to standard error. **=*P*<0.001 compared to control (mouse IgG)

PC3 binding to BME. The antibody directed against β1 integrin significantly inhibited ($P$ = 0.000003) PC3 binding by 64%. None of the other antibodies studied showed any marked reduction on the epithelial:endothelial cell adhesion.

Due to the interpatient heterogeneity observed in prostate cancer (George, 1988), assays were repeated with 11 primary human epithelial cell samples from patients with CaP or BPH. Our previous experiments demonstrated very little binding of primary cells (isolated from CaP or BPH tissue) after 1 h; maximum binding was seen at 24 h (Lang et al, 1997). Although this difference was lost after one passage in culture (data not shown). We therefore used 24 h as time point for subsequent experiments. Primary epithelial cells from patients with CaP showed significantly greater adhesion to BME and BMS compared to HUVEC ($P$ = 0.00007 and $P$ = 0.00003) (Figure 5A). The same was also found for epithelia from BPH patients, $P$ = 0.00002 and $P$ = 0.008 (Figure 5B). Note that the large error bars are a reflection of patient heterogeneity, however, all patients followed the same binding trend.

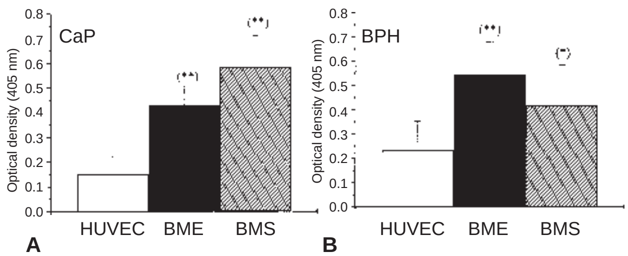

**Figure 5** Binding of epithelial cells (5 × 10⁴ per well) isolated from patients with CaP ($n$ = 11) to endothelium or stroma (**A**) and BPH ($n$ = 11) (**B**). Adhesion was allowed to take place for 24 h. Each patient sample was measured in 3 individual experiments (3 replicas per assay) and the average value calculated. Data represent the mean plus standard errors for the combined data from 11 patients. *= $P$<0.05 and **=$P$<0.001 (compared to HUVEC)

### Invasion assay

The ability of prostate epithelial cells (PNT2-C2, PC3 and epithelial cells derived from patients with BPH) to invade through matrigel invasion chambers in response to endothelial or BMS cultures was assessed. Indirect co-culture of PNT2-C2 cells with endothelium or stroma led to no significant increase in their invasive ability compared to tissue culture plastic alone. The metastatic cell line, PC3, showed an increase in invasion in response to BME (231 cells/average field of view) and BMS (136 cells/average field of view) but not to HUVEC (15 cells/average field of view) compared to tissue culture plastic (16 cells/average field of view). The increase in invasion in response to BME was significantly greater than observed with BMS ($P$ = 0.0437). None of the BPH epithelial cells tested ($n$ = 3) demonstrated any invasive ability (Figure 6).

### DISCUSSION

We have developed simple models to study the interactions of prostatic epithelial cells with endothelium or stromal layers. These assays have enabled us to establish that malignant and non-malignant prostate epithelial cells preferentially adhere to bone marrow endothelium at similar levels. Lehr and Pienta (1998) also reported a 3-fold increase in binding to bone marrow endothelial cells (albeit a cell line) compared to HUVEC. However, only the malignant PC3 cells demonstrated increased invasion in response to bone marrow endothelium. PC3 cells bound not only maximally but their rate of binding was significantly faster to bone marrow endothelium compared to bone marrow stroma and HUVEC. PC3 cells bound to a lung endothelium at an intermediate level between HUVEC and bone marrow endothelium. As the lung is the third commonest site for prostate metastases after lymph nodes and bone marrow (Jacobs, 1983) our in vitro model appears to show a similar pattern of metastatic selectivity to that found in vivo. It would be of interest to expand our studies in the future to include primary endothelial cells isolated from different organs such as liver, kidney and brain.

Human umbilical vein endothelial cells have been used in several studies to look at tumour cell binding to endothelium (Dejana et al, 1992; Majuri et al, 1992; Merwin et al, 1992; Iwai

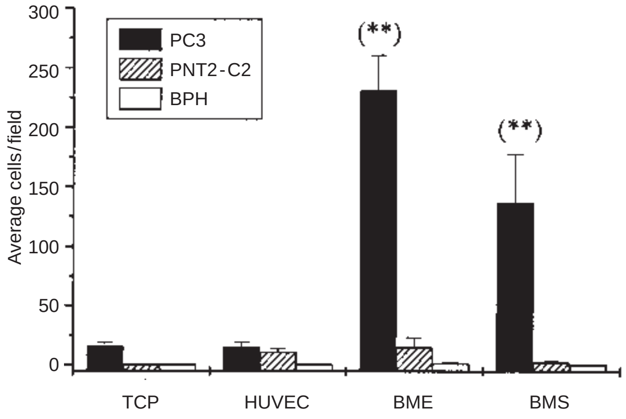

**Figure 6** Invasion of prostate epithelial cells (PC3, PNT2-C2 or BPH) through matrigel-coated invasion chambers in response to indirect HUVEC, BME, BMS or tissue culture plastic (TCP). The data represent mean plus standard error bars generated from 3 experiments (3 replicas per assay). **= $P$ < 0.001 (compared to binding to HUVEC)

© 2001 Cancer Research Campaign et al, 1993; Takada et al, 1993; Zaifert and Cohen, 1993; Price et al, 1996; Kannagi, 1997). However, there are phenotypic and functional differences between endothelial cells derived from large vessels and those of the microvasculature (McCarthy et al, 1991). Since tumour cell extravasation occurs generally within the microvasculature (Alby and Auerbach, 1984) using large vessel endothelium (such as HUVEC) may not be the best physiological cell type to use as a model. Consequently, it is important to look at tumour cell interactions with endothelium from their preferred metastatic site in order to unravel the mechanism involved in the process of metastasis.

We have shown that the integrin β1 plays an important role in the attachment of PC3 cells to bone marrow endothelium. Increased expression of β1 has been linked with higher grades of prostate cancer (Murant et al, 1997). Unlike the data observed with PC3 binding to bone marrow stroma, antibodies against α2 did not prevent PC3 binding to bone marrow endothelial cells. Antibodies against β4, α4, α5, sialy Lewis X, CD31, P selecting and VCAM-1 also demonstrated no inhibitory affects on adhesion. Jorgensen et al (1995) have linked the up-regulation of the oligosaccharide sialy Lewis X with metastatic prostate cancer. However, a study by Martensson and colleagues (Martensson et al, 1995) showed strong expression of Lewis Y antigens in 26 out of 30 patients with prostate cancer and only 5 showed non-sialy Lewis X. Benign tissue was negative for Lewis Y and only the occasional cell was positive for Lewis X. Our results indicate that sialy Lewis X is not involved in the binding of the PC3 cell line to bone marrow endothelium. Whether this will also be the case for primary prostatic epithelial cells has yet to be established. These inhibition data are conflicting to those reported by Lehr and Pienta (1998) who found no inhibition using a β1 antibody in the binding of PC3 cells to a bone marrow endothelial cell line. They suggested a role for galectin-3, a galactose-binding molecule, in PC3 binding. Since we have not directly compared primary bone marrow endothelial cells to their cell line the reasons for these differences remain unknown. However, change of cell phenotype during prolonged culture is a known and common feature. Changes in adhesion molecule expression between immortalized and primary bone marrow endothelial cells could therefore account for this discrepancy. Another possibility is variations in commercial antibodies that recognize different epitopes, which may or may not inhibit cell adhesion in the assays used.

Lectin:carbohydrate interactions may be involved in forming a loose association to bone marrow endothelium with integrins playing a more crucial role in securing strong adhesion. CD31 has been shown to amplify β1-mediated adhesion to endothelial cells (Tanaka et al, 1992). We found no inhibitory effects on PC3 binding to endothelial cells using a CD31-blocking antibody. A synergistic effect could possibly occur by using anti-CD31 and anti-β1 in combination. Price et al (1996) have demonstrated that pre-treatment of a breast adenocarcinoma cell line and a melanoma cell line with antibodies against β1 integrin substantially inhibited their adhesion to HUVEC. They also showed that simultaneous treatment of tumour and endothelial cells produced an additive-blocking effect. A limited number of antibodies have been used so far in these studies, other integrins such as α3, α6, β3, αvβ3 or αIIbβ3 may yet be found to be involved in prostate epithelial cell interactions with bone marrow endothelial cells. Indeed, αIIbβ3 has recently been associated with prostate cancer metastasis in a study of prostatic cell lines (Trikha et al, 1998). In addition, the PC3 cell line has been shown to express αvβ3 and 16/16 primary CaP epithelial cells. Interestingly this adhesion molecule was not found on normal prostatic epithelial cells (Zheng et al, 1999).

It has been reported that there are differences in integrin expression between primary and metastatic prostate epithelial cells with increased levels of β1, α2 and α6 being associated with higher histological grades (Bonkhoff et al, 1993; Murant et al, 1997). Variations in integrin expression are observed for different human prostate cell lines in particular none expressed α4β1 and differences in α2β1 and α3β1 were observed (Rokhlin and Cohen, 1995). Also, we have found previously that there are variations in the levels of inhibition of binding of prostate epithelial cells with antibodies against α3 and α5 to bone marrow stroma (Lang et al, 1997). It is likely therefore that similar variations may be observed when looking at binding to primary bone marrow endothelium. Such differences are likely to be more relevant clinically than data obtained using cell lines.

For this reason we also thought it important to use primary prostate samples in our experiments. The binding of primary prostatic epithelial cells derived from CaP or BPH patients closely mimicked that seen with PC3 cells, but the non-malignant PNT2-C2 cell line was substantially less invasive in response to BME than the metastatic cell line, PC3. In agreement none of the 3 BPH samples tested demonstrated any invasive ability. Subtle differences may exist which affect the ability of these cells to extravasate though a bone marrow endothelial barrier. Of the cells that bind to bone marrow endothelium it is not known how many would be capable of subsequent metastases formation.

No differences were observed in the binding behaviour of PNT2-C2 and PC3 or BPH and CaP prostatic epithelial cells to BME. These results suggest that any prostate epithelial cell entering the circulation has the potential to adhere to bone marrow endothelium. Clearly, in vivo, not all circulating prostatic epithelial cells adhere to endothelium and go on to invade and form secondary tumours. There is evidence to support this as large numbers of epithelial cells are released into the circulation during prostatic resection without any adverse effects on the patients overall survival (Arcangeli et al, 1995). Our recent data suggest therefore that the invasive nature of malignant versus non-malignant epithelial cells may be a critical step in the formation of metastasis. It is essential to explore these differences further since both BPH and CaP cells show growth stimulation in response to bone marrow stroma (Lang et al, 1998). Using the cellular models described here we hope to study the molecular nature of the interaction of prostate epithelia and bone marrow endothelia, in particular the changes required for invasion.

### ACKNOWLEDGEMENTS

Work supported by the Association for International Cancer Research, grant number 98/27. NGT is supported by the Cancer Research Campaign and SHL is supported by Yorkshire Cancer Research. The authors thank Mrs Claire Hart for her excellent technical assistance.

### REFERENCES

Alby L and Auerbach R (1984) Differential adhesion of tumor cells to capillary endothelial cells in vitro. *Proc Natl Acad Sci USA* **81**: 5739–5743

Arcangeli G, Micheli A, Verna L, Saracino B, Arcangeli G, Giovinazzo GD, Angelo L, Pansadoro V and Sternberg CN (1995) Prognostic impact of transurethral resection on patients irradiated for localized prostate cancer. *Radiother Oncol* **35**: 123–128

 © 2001 Cancer Research Campaign

Auerbach R, Lu WC, Pardon E, Gumkowski F, Kaminska G and Kaminska M (1987) Specificity of adhesion between murine tumor cells and capillary endothelium: an in vitro correlate of preferential metastasis in vivo. *Cancer Res* **47**: 1492–1496

Berthon P, Cussenot O, Hopwood L, Le Duc A and Maitland NJ (1995) Functional expression of SV 40 in normal human prostatic epitheial and fibroblastic cells: Differentiation pattern of non-tumorigenic cell lines. *Int J Oncology* **6**: 333–343

Body JJ (1992) Metastatic bone disease: clinical and therapeutic aspects. *Bone* **13** Suppl 1: S57–62

Bonkhoff H, Stein U and Remberger K (1993) Differential expression of alpha 6 and alpha 2 very late antigen integrins in the normal, hyperplastic, and neoplastic prostate: simultaneous demonstration of cell surface receptors and their extracellular ligands. *Hum Pathol* **24**: 243–248

Coutinho LH, Gilleece MH, De Wynter EA, Will A and Testa NG (1993) Colonal and long-term cultures using human bone marrow. In: *Haemopoiesis: A Practical Approach* Testa NG and Molineux G (eds) pp. 75–106. IRL Press: Oxford, UK

Dejana E, Martin Padura I, Lauri D, Bernasconi S, Bani MR, Garofalo A, Giavazzi R, Magnani J, Mantovani A and Menard S (1992) Endothelial leukocyte adhesion molecule-1-dependent adhesion of colon carcinoma cells to vascular endothelium is inhibited by an antibody to Lewis fucosylated type I carbohydrate chain. *Lab Invest* **66**: 324–330

Fidler IJ, Gersten DM and Hart IR (1978) The biology of cancer invasion and metastasis. *Adv Cancer Res* **28**: 149–250

Galasko CS (1981) Bone metastases studied in experimental animals. *Clin Orthop* **155**: 269–285

George NJ (1988) Natural history of localised prostatic cancer managed by conservative therapy alone. *Lancet* **1**: 494–497

Honn KV and Tang DG (1992) Adhesion molecules and tumor cell interaction with endothelium and subendothelial matrix. *Cancer Metastasis Rev* **11**: 353–375

Iwai K, Ishikura H, Kaji M, Sugiura H, Ishizu A, Takahashi C, Kato H, Tanabe T and Yoshiki T (1993) Importance of E-selectin (ELAM-1) and sialyl Lewis(a) in the adhesion of pancreatic carcinoma cells to activated endothelium. *Int J Cancer* **54**: 972–977

Jackson CJ, Garbett PK, Nissen B and Schrieber L (1990) Binding of human endothelium to Ulex europaeus I-coated Dynabeads: application to the isolation of microvascular endothelium. *J Cell Sci* **96**: 257–262

Jacobs SC (1983) Spread of prostatic cancer to bone. *Urology* **21**: 337–344

Jensen OM, Esteve J, Moller H and Renard H (1990) Cancer in the European Community and its member states [published erratum appears in Eur J Cancer 1991; 27(12):1717]. *Eur J Cancer* **26**: 1167–1256

Jorgensen T, Berner A, Kaalhus O, Tveter KJ, Danielsen HE and Bryne M (1995) Up-regulation of the oligosaccharide sialyl Lewis X: a new prognostic parameter in metastatic prostate cancer. *Cancer Res* **55**: 1817–1819

Kannagi R (1997) Carbohydrate-mediated cell adhesion involved in hematogenous metastasis of cancer. *Glycoconj J* **14**: 577–584

Kostenuik PJ, Sanchez-Sweatman O, Orr FW and Singh G (1996) Bone cell matrix promotes the adhesion of human prostatic cells via $\alpha 2\beta 1$ integrin. *Clin Exp Metastasis* **14**: 19–26

Lang SH, Clarke NW, George NJ and Testa NG (1997) Primary prostatic epithelial cell binding to human bone marrow stroma and the role of alpha2beta1 integrin. *Clin Exp Metastasis* **15**: 218–227

Lang SH, Clarke NW, George NJ, Allen TD and Testa NG (1998) Interaction of prostate epithelial cells from benign and malignant tumor tissue with bone-marrow stroma. *Prostate* **34**: 203–213

Lehr JE and Pienta KJ (1998) Preferential adhesion of prostate cancer cells to a human bone marrow endothelial cell line. *J Natl Cancer Inst* **90**: 118–123

Majuri ML, Mattila P and Renkonen R (1992) Recombinant E-selection-protein mediates tumor cell adhesion via sialyl-Le(a) and sialyl-Le(x). *Biochem Biophys Res Commun* **182**: 1376–1382

Martensson S, Bigler SA, Brown ML, Lange PH, Brawer MK and Hakomori S (1995) Sialy-Lewis(x) and related carbohydrate antigens in the prostate. *Hum Pathol* **26**: 735–739

Masek LC and Sweetenham JW (1994) Isolation and culture of endothelial cells from human bone marrow. *Br J Haematol* **88**: 855–865

McCarthy SA, Kuzu I, Gatter KC and Bicknell R (1991) Heterogeneity of the endothelial cell and its role in organ preference of tumour metastasis. *Trends Pharmacol Sci* **12**: 462–467

Merwin JR, Madri JA and Lynch M (1992) Cancer cell binding to E-selectin transfected human endothelia. *Biochem Biophys Res Commun* **189**: 315–323

Murant SJ, Handley J, Stower M, Reid N, Cussenot O and Maitland NJ (1997) Co-ordinated changes in expression of cell adhesion molecules in prostate cancer. *Eur J Cancer* **33**: 263–271

Nagle RB, Knox JD, Wolf C, Bowden GT and Cress AE (1994) Adhesion molecules, extracellular matrix and proteases in prostate carcinoma. *J Cell Biochem* **26**: 232–237

Nicolson GL and Winkelhake JL (1975) Organ specificity of blood-borne tumour metastasis determined by cell adhesion? *Nature* **255**: 230–232

Paget S (1989) The distribution of secondary growths in cancer of the breast. 1889 [classical article]. *Cancer Metastasis Rev* **8**: 98–101

Price EA, Coombe DR and Murray JC (1996) beta-1 Integrins mediate tumour cell adhesion to quiescent endothelial cells in vitro. *Br J Cancer* **74**: 1762–1766

Rokhlin OW and Cohen MB (1995) Expression of cellular adhesion molecules on human prostate tumor cell lines. *Prostate* **26**: 205–212

Shevrin DH, Gorny KI and Kukreja SC (1989) Patterns of metastasis by the human prostate cancer cell line PC-3 in athymic nude mice. *Prostate* **15**: 187–194

Soligo D, Shiro R, Luksch R, Mannara G, Quirici N, Parravicini C and Lambertenghi DG (1990) Expression of integrins in human bone marrow. *Br J Haematol* **76**: 323–332

Takada A, Ohmori K, Yoneda T, Tsuyuoka K, Hasegawa A, Kiso M and Kannagi R (1993) Contribution of carbohydrate antigens sialyl Lewis A and sialyl Lewis X to adhesion of human cancer cells to vascular endothelium. *Cancer Res* **53**: 354–361

Tanaka Y, Albelda SM, Horgan KJ, van Seventer GA, Shimizu Y, Newman W, Hallam J, Newman PJ, Buck CA and Shaw S (1992) CD31 expressed on distinctive T cell subsets is a preferential amplifier of beta 1 integrin-mediated adhesion. *J Exp Med* **176**: 245–253

Trikha M, Raso E, Cai Y, Fazakas Z, Paku S, Porter AT, Timar J and Honn KV (1998) Role of alphaII(b)beta3 integrin in prostate cancer metastasis. *Prostate* **35**: 185–192

Zaifert K and Cohen MC (1993) COLO 205 utilizes E-selection to adhere to human endothelium. *Clin Immunol Immunopathol* **68**: 51–56

Zetter BR (1990) The cellular basis of site-specific tumor metastasis. *N Engl J Med* **322**: 605–612

Zheng DQ, Woodard AS, Fornaro M, Tallini G and Languino LR (1999) Prostatic carcinoma cell migration via alpha(v)beta3 integrin is modulated by a focal adhesion kinase pathway. *Cancer Res* **59**: 1655–1664

© 2001 Cancer Research Campaign 
